# Identification and analysis of odor-active compounds from *Choerospondias axillaris* (Roxb.) Burtt et Hill with different moisture content levels and lacquer treatments

**DOI:** 10.1038/s41598-020-71698-0

**Published:** 2020-09-09

**Authors:** Qifan Wang, Jun Shen, Bin Zeng, Huiyu Wang

**Affiliations:** Key Laboratory of Bio-Based Material Science and Technology, Ministry of Education, Northeast Forestry University, 26 Hexing Road, Harbin, 150040 China

**Keywords:** Health care, Risk factors

## Abstract

The problem of indoor odors can greatly affect a room’s occupants. To identify odorants and comprehensively evaluate emissions from wooden materials, emissions and odors from *Choerospondias axillaris* (Roxb.) Burtt et Hill with different moisture content percentages and lacquer treatments were investigated in this study. Thermal desorption–gas chromatography–mass spectroscopy/olfactometry was used to analyze the release characteristics. In total, 11 key odor-active compounds were identified as moisture content gradually decreased, concentrating between 15 and 33 min. Total volatile organic compounds, total very volatile organic compounds, and total odor intensity decreased as moisture content decreased. In addition, 35 odor-active compounds, including aromatics, alkenes, aldehydes, esters, and alcohols, were identified in the odor control list. Polyurethane (PU), ultraviolet (UV), and waterborne coatings had a good inhibitory effect on eight odor characteristics, but some scents arose after lacquer treatment. For equilibrium moisture content, the major characteristics of *Choerospondias axillaris* were fragrant (9.4) and mint-like (3.0) compared with the fragrant (8.2), fruity (7.8), and pleasant (5.8) characteristics of PU coating; the flowery (5.9), fragrant (5.0), glue-like (4.3), and pineapple-like (4.3) characteristics of UV coating; and the antiseptic solution (3.6), fragrant (2.9), cigarette-like (2.8), and fruity (2.5) characteristics of waterborne coating. Based on multicomponent evaluation, a *Choerospondias axillaris* board with waterborne coating was suggested for use indoors.

## Introduction

Compared with wood-based panels, solid-wood furniture and products have the natural, ecofriendly characteristics with less resin consumption during the manufacturing process. However, during indoor use, wooden furniture and materials can release hazardous substances into the surrounding environment because of inevitable lacquer treatment^1^. Numerous studies have researched volatile organic compounds (VOCs) in the field of wooden materials. Most studies have focused on the sampling and analysis method^2–4^, decorative properties^5,6^, release limit standard^7^, release characteristics^8^, and impact of environmental conditions^9,10^. However, the odor problem follows emission from materials, which may annoy a room’s occupants and impair their health. Therefore, odor testing is important in the research of emissions from materials^11^.

Wood primarily comprises the biopolymers cellulose, hemicellulose, and lignin, with minor amounts of inorganic compounds and extractives^12^. Most odorants come from the extractives, which are located in the resin duct, gum duct, and parenchyma cells and include aliphatic compounds, terpenes, terpenoids, and phenols. Related research showed the content of these compounds varies with tree species, sampling position, and origin. In addition, with change in the chemical composition of wood under conditions of high temperature, high humidity, and high pressure, odors were produced. For example, the degradation products of hemicellulose under high temperature and high humidity were formic acid, acetic acid, and propionic acid^13^. Odors also may be produced by the metabolism or degradation of starch or other wood carbohydrates with microorganisms in the wood.

Human olfaction has long evolutionary history; chemical senses for chemical compounds interacting with receptor molecules are the stimulus prerequisites for eliciting olfactory sensations^14^. The odor characteristics of volatile molecules are related to their reversible low energy binding to protein receptors. The binding specificity of these receptors depends on the actual topography of protein receptors, which is still unknown. The binding energy is determined by van der Waals force, hydrogen bond, and hydrophobic binding^15^. In aspects of odor research of wooden materials, gas chromatography–mass spectroscopy/olfactometry (GC–MS/O) has been widely used in odor analysis^16^. The odorants in the wood of *Calocedrus decurrens* (Torr.) Florin was explored, and more than 60 odorous substances and 22 most potent odorants were successfully identified. The study found the main odorants were a series of terpenes, several degradation products of fatty acids, and several odorants with a phenolic core moiety^17^. The influence of melamine-impregnated paper on the odor emissions of medium density fiberboard (MDF) was investigated by Li et al*.* who found the melamine-impregnated paper could inhibit the mass concentration and odor intensity of odor characteristic compounds^18^. The odors emitted from particleboard with alkyd resin enamel under different environments were studied by Wang et al*.*^19^ They found that total volatile organic compound (TVOC) concentration and total odor intensity increased as temperature and relative humidity increased but that they decreased as the ratio of air exchange rate to loading factor increased. Temperature had a greater effect on the release of VOCs.

*Choerospondias axillaris* (a deciduous broad-leaved tree species in the Anacardiaceae family), an excellent fast-growing, artificial-forest species in South China^20^, was investigated in this study. For its characteristics of hard texture, temperature and water resistance, unease deformation, and beautiful natural texture, it is mainly used in solid-wood furniture, as well as in carriages, boat boards, wharf engineering, construction, board materials, gunstocks, agricultural tools, musical instruments, handicrafts, and others^21^. In this study, the odors released from *Choerospondias axillaris* under different percentages of moisture content was investigated by using a microchamber combined with thermal desorption–gas chromatography–mass spectroscopy/olfactometry (TD–GC–MS/O). The effect of different lacquer treatments on emissions was explored, and the odor characteristics were compared. Multicomponent evaluation method was used to evaluate the influence of *Choerospondias axillaris* with different lacquer treatments. Besides the VOCs, very volatile organic compounds (VVOCs) within retention below C6 were also taken into account, as pointed out by German Committee for Health-Related Evaluation of Building Products (AgBB)^22,23^.

## Results and discussion

### Effect of moisture content on odor emissions from *Choerospondias axillaris*

**Figure 1:**
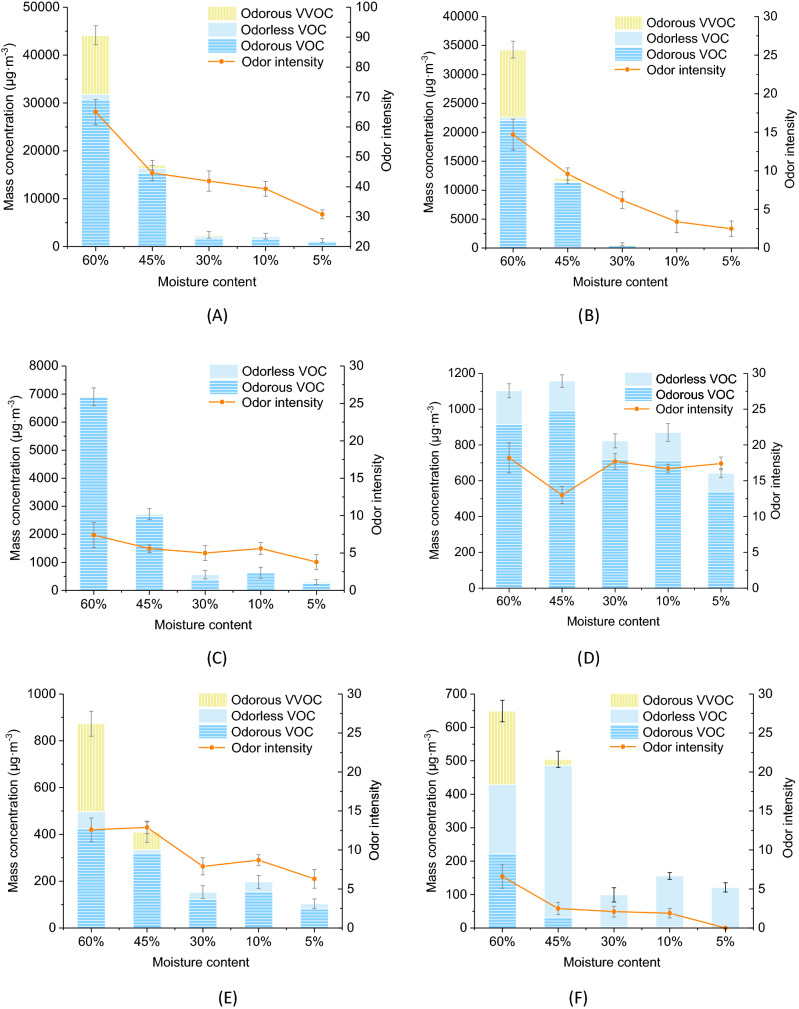
VOC and VVOC concentrations of different components and total odor intensity from *Choerospondias axillaris* under different percentages of moisture content. (**A**) Components. (**B**) Alcohols. (**C**) Alkenes. (**D**) Aromatics. (**E**) Aldehydes and ketones. (**F**) Esters.

To explore the effect of moisture content on emissions and odors from *Choerospondias axillaris*, the release characteristic with different percentages of moisture content was investigated. In general, the main components can be classified as aromatics, alkenes, aldehydes and ketones, esters, and alcohols. alkanes and other components (in small amounts) were also found. The mass concentration of different components and total odor intensity are shown in Fig. 1.

The TVOC concentration was significantly higher than the total very volatile organic compound (TVVOC) concentration throughout the decline process of moisture content, as well as for the different components that belong to VOCs and VVOCs. Alcohols and alkenes were the main release components of *Choerospondias axillaris*, followed by aromatics, aldehydes and ketones, and esters. The main components of VOCs were aromatics, alkenes, aldehydes and ketones, esters, alcohols, whereas those of VVOCs were mainly from aldehydes and ketones, esters, and alcohols. Nearly all VVOCs detected in this studied have odor characteristics except some alcohols (32 µg/m^3^); in addition, more than 70% of TVOCs have odor profiles throughout the change process of moisture content.

The moisture content had a significant effect on VOC and VVOC release from *Choerospondias axillaris*. When the moisture content is 60%, TVOC, TVVOC, and Odor Intensity (OI) reached their maximum values. TVOC, TVVOC, and OI rates decreased as the moisture content decreased. The effect on TVOC and TVVOC was more significant when the moisture content dropped from 60 to 30% and was reduced after 30%. The percentages of decline from 60 to 30% moisture content were 93.28% and 97.67%, respectively, whereas TVOC and TVVOC declined at 15,460 µg/m^3^ (48.60%) and 11,643 µg/m^3^ (94.44%) from 60 to 45% moisture content and 14,215 µg/m^3^ (86.92%) and 398 µg/m^3^ (58.05%) from 45 to 30% moisture content. Below 30% moisture content, the rate of decline for total release began to slow. From 30 to 10% moisture content, the concentration of VOCs and VVOCs decreased to 72 and 177 µg/m^3^ and then continued to drop (from 10 to 5% moisture content) to 843 and 83 µg/m^3^, respectively. The composition emissions and odors from wood were directly related to the movement of water in the wood. With the decrease in moisture content, VOCs and VVOCs were released from the wood through evaporation and migration of water, leading to diminished concentrations^24^. As the average value of the fiber saturation point, 30% is the turning point for the influence of moisture content on emissions from *Choerospondias axillaris*. In this state, the adsorbed water in the cell walls of the wood is saturated, but there is no free water in the cell cavities and cell gaps. The result showed that the change of free moisture content had a great influence on the release components of wood. When the moisture content is below the fiber saturation point, the movement of absorbed water is subdivided into two parts: diffusion transfer because of the vapor pressure gradient and moisture movement caused by pressure fluctuation because of the variation of the medium. In this stage, the influence of moisture content on emissions diminished. A similar phenomenon could be found in alcohols and in aldehydes and ketones, which TVOC and TVVOC decrease more quickly from 60 to 30% moisture content. TVOC and TVVOC of alcohols reduced to 22,214 µg/m^3^ (98.22%) and 11,438 µg/m^3^ (98.00%) in this process, whereas TVOC of aldehydes and ketones fell to 325 µg/m^3^ (65.43%) and TVVOC of aldehydes and ketones fell to zero. For alkenes, TVOC also showed the fastest descent rate from 60 to 30% moisture content (6,339 µg/m^3^, 91.85%), and no VVOC was found in this group. TVVOC of esters decreased from 220 to 0 µg/m^3^ from 60 to 30% moisture content, TVOC began to drop after 45% moisture content. For aromatics, no VVOCs were detected, and the change of TVOC with moisture content was irregular.

**Figure 2:**
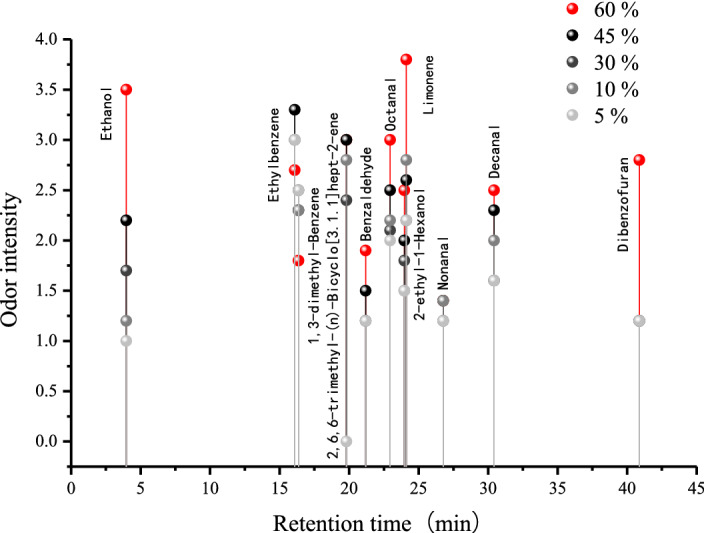
Odor intensity-time diagram of 11 key odor characteristic compounds of *Choerospondias axillaris* with different percentages of moisture content.

The OI of TVOC and TVVOC showed a significant rate of decline when the moisture content changed from 60 to 45% (decrease of 20.40) and 10% to 5% (decrease of 8.50). At moisture content of 60%, 45%, 30%, 10%, and 5%, the values were 65.00, 44.60, 41.90, 39.30, and 30.80, respectively. Moisture content influences odor emissions through its effect on soluble extractives. With decreased moisture content, the amounts of soluble extractives were reduced and resulted in a decrease of odorants^25^. From 60 to 5% moisture content, the OI for alcohols declined almost linearly from 14.7 to 2.5, and the OI of esters decreased from 6.6 to 0. The OI of alkenes and of aldehydes and ketones decreased slightly with the decrease in moisture content, whereas the OI of aromatics had no obvious regularity. In total, 11 key odor-active compounds were identified as moisture content decreased gradually, concentrating between 15 and 33 min in gas chromatography–olfactometry (GC–O) (Fig. 2). The odor intensity of 8 odor-active compounds decreased with the decrease in moisture content. Among them, the odor intensity of benzaldehyde, dibenzofuran, octanal, ethanol, and 2-ethyl-1-hexanol decreased steadily with the decrease in moisture content, reducing 0.7, 1.6, 1.0, 2.5, and 1, respectively. The intensity of 2,6,6-trimethyl-(ñ)-bicyclo[3.1.1]hept-2-ene, limonene, and decanal still presented a downward trend with the decrease in moisture content, despite the odor intensity fluctuating when the moisture content decreased from 30 to 10%. This phenomenon may occur because the mass concentration of these three key odor-active compounds increased temporarily when the moisture content decreased from 30 to 10% and then continued to decrease. Related reports showed the mass concentration can affect the odor intensity for certain types of compounds^26,27^, which was also reported by Weber–Fechner’s law^28^, showing the odor intensity is logarithmically related to odorant concentration, which can be calculated with the following equation:

1 $$I = K\log C$$

where *I* is the odor intensity, *K* is a constant, and *C* is the mass concentration of the odorant.

### Characterization of odor-active substances from *Choerospondias axillaris* with different lacquer treatments

The key odor-active compounds were characterized as potent smell contributors for comparative analysis of mass spectral data, reference to relevant odor literature, and the parameter retention index^29^. According to the factors of the compounds’ *R*_*i*_ and odor intensity, the odor control list was determined. The odorous substance was recorded when the odor intensity of any of the four samples was greater than 1 or *R*_*i*_ was greater than 0.01. In total, 35 odor-active compounds were identified in the odor control list. The odor characteristics and relevant parameters of *Choerospondias axillaris* with different lacquer treatments are shown in Table 1. The key odorous components of this material were primarily from aromatics, alkenes, aldehydes, esters, and alcohols. Alkane was not detected by key odor-active compounds, which was similar to the results found by Félix et al*.*^30^.

**Table 1:** Odor control list of *Choerospondias axillaris* with different lacquer treatments.

	Nos.	Compounds	LCI	RI	Formula	Compound’s odor character, intensity and R*i* (C*i*/LCI*i*)			
						None	PU	UV	Water
Aromatics	1	Toluene	2,900	765	C_7_H_8_	(Aromatic, 1.6)/0.0512	(Aromatic, 1.2)/0.0116	(Aromatic, 1.6)/0.0472	(Aromatic, 1.2)/0.0092
	2	o-Xylene	500	861	C_8_H_10_	(Aromaitc, 0.8)/0.1054	/	/	/
	3	Ethylbenzene	850	868	C_8_H_10_	(Aromatic, 3)/0.0715	(Aromatic, 3)/0.0659	(Aromatic, 2.7)/0.0301	(Aromatic, 1.7)/0.0173
	4	1,3-Dimethyl-Benzene	500	877	C_8_H_10_	(Metal-like, 2.3)/0.3772	/	(Metal-like, 1.9)/0.1475	(Metal-like, 1.6)/0.0904
	5	p-Xylene	500	899	C_8_H_10_	/	(Aromatic, sweety, 1.8)/0.397	(Aromatic, sweety, 0.7)/0.0167	/
	6	Benzaldehyde	430	976	C_7_H_6_O	(Almond-like, 1.2)/0.0697	/	(Almond-like, 0)/0.0135	(Almond-like, 0)/0.013
	7	1,2,4-Trimethyl-Benzene	450	982	C_9_H_12_	/	(Aromatic, 0.6)/0.0208	/	/
	8	1,2,3-Trimethyl-Benzene	450	1,007	C_9_H_12_	/	(Orchid candy-like, 2.6)/0.0503	/	/
	9	Acetophenone	490	1,088	C_8_H_8_O	(Sweety, 0.8)/0.0387	/	(Sweety, 0.3)/0.0105	/
	10	1-Methylene-1*H*-Indene	496	1,237	C_10_H_8_	(Wood-like, 2)/0.0324	/	(Wood-like, 1.7)/0.012	/
	11	Butylated Hydroxytoluene	100	1,560	C_15_H_24_O	(Cooling mint-like, 1)/0.3619	(Musty, 0.5)/0.0339	/	/
	12	Dibenzofuran	/	1592	C_12_H_8_O	(Mixture, 1.2)/–	/	(Mixture, 0)/–	/
Alkenes	13	2,6,6-Trimethyl-(ñ)-Bicyclo[3.1.1]hept-2-ene	1,400	948	C_10_H_16_	(Pine-like, pungent, 2.8)/0.0224	/	/	/
	14	Limonene	5,000	1,051	C_10_H_16_	(Fresh lemon peel-like, pleasant, 2.8)/0.1185	/	/	/
	15	Copaene	1,400	1,468	C_15_H_24_	/	/	/	(Nut-like, 2.3)/0.0034
Aldehyde ketone	16	Hexanal	900	804	C_6_H_12_O	(Green grassy fruity, 1.6)/0.0265	(Fragrant, 1.6)/0.0069	/	/
	17	Octanal	900	1,017	C_8_H_16_O	(Fruity, sour, 2.2)/0.0221	/	(Fruity, sour, 2)/0.0087	/
	18	Nonanal	900	1,127	C_9_H_18_O	(Citrus-like, 1.4)/0.047	(Citrus-like, 1)/0.0101	(Citrus-like, 1)/0.0131	(Citrus-like, 1)/0.01
	19	Decanal	900	1,255	C_10_H_20_O	(Fresh mint-like, 2)/0.067	(Fresh mint-like, 1.5)/0.0091	(Fresh mint-like, 1.3)/0.0217	(Fresh mint-like, 1.5)/0.0107
Esters	20	Ethyl Acetate	3,620	616	C_4_H_8_O_2_	/	(Fruity, 2.7)/0.0685	(Irritating, glue-like, pineapple-like, fragrant, 4.3)/1.6593	(Fruity, 2.5)/0.0502
	21	Acetic acid, 2-methylpropyl ester	4,800	773	C_6_H_12_O_2_	/	(Fruit-like, flowery, fresh, 2.6)/0.0026	/	/
	22	Acetic acid, butyl ester	4,780	819	C_6_H_12_O_2_	/	(Fruity, 2.5)/0.0768	(Fruity, 0.9)/0.004	/
	23	2-Pentanol, acetate	5,380	855	C_7_H_14_O_2_	/	(Fragrant, pleasant, 2.5)/0.005	(Fragrant, 0)/0.0006	/
	24	1-Methoxy-2-propyl acetate	2,700	873	C_6_H_12_O3	/	(Sweety, 1.2)/0.0424	(Sweety, 0)/0.0012	/
	25	Pentanedioic acid, dimethyl ester	50	1,156	C_7_H_12_O_4_	/	(Pleasant, 2)/0.2592	/	/
	26	2-Methyl-Propanoic acid,1-(1,1-dimethylethyl)-2-methyl-1,3-propanediyl ester	/	1653	C_16_H_30_O_4_	(Antiseptic solution, 1.9)/–	(Antiseptic solution, 2.3)//	(Antiseptic solution, 2.3)/–	(Antiseptic solution, 3.6)/–
Alcohols	27	Ethanol	1,860	< 600	C_2_H_6_O	(Fragrant, 1.2)/0.0262	(Alcohol-like, 1.5)/0.1073	(Alcohol-like, 2.5)/0.3489	(Alcohol-like, 1.4)/0.0584
	28	2-Butanol	170	607	C_4_H_10_O	/	(Pleasant, 0.7)/0.0561	/	/
	29	1-Butanol	3,000	663	C_4_H_10_O	/	(Alcohol-like, 1.1)/0.0164	(Alcohol-like, 0.8)/0.0068	(Alcohol-like, 0.8)/0.0056
	30	1-Methoxy-2-Propanol	120	675	C_4_H_10_O_2_	/	(Mild, pleasant, 0.6)/0.0552	/	/
	31	2-Pentanol	200	704	C_5_H_12_O	/	(Wine-like, 0.4)/0.0277	/	/
	32	2-Ethyl-1-Hexanol	300	1,045	C_8_H_18_O	(Flowery, 1.5)/0.0905	/	(Flowery, 1.6)/0.0499	(Flowery, 1.2)/0.0252
	33	2-(2-hydroxypropoxy)-1-Propanol	7,200	1,324	C_6_H_14_O_3_	/	/	/	(Cigarette-like, 2.8)/0.0109
Acid	34	Acetic acid	1,200	< 600	C_2_H_4_O_2_	(Sour, 1)/0.0142	(Sour, 0.9)/0.0072	/	(Sour, 0.5)/0.0041

The key odor-active compounds (odorants with an odor intensity greater than 2) of four boards were identified. Seven key odor-active compounds were detected from *Choerospondias axillaris* with no treatment, namely, ethylbenzene, limonene, 2,6,6-trimethyl-(ñ)-bicyclo[3.1.1]hept-2-ene, 1,3-dimethyl-benzene, octanal, decanal, and 1-methylene-1H-indene. For *Choerospondias axillaris* with polyurethane (PU), eight key odor-active compounds were found: ethylbenzene; ethyl acetate; acetic acid, 2-methylpropyl ester; 1,2,3-trimethyl-benzene; 2-pentanol, acetate; acetic acid, butyl ester; 2-methyl-propanoic acid, 1-(1,1-dimethylethyl)-2-methyl-1,3-propanediyl ester; and pentanedioic acid, dimethyl ester. For ultraviolet (UV)-coated *Choerospondias axillaris*, five key odor-active compounds were found: ethyl acetate; ethylbenzene; ethanol; 2-methyl-propanoic acid, 1-(1,1-dimethylethyl)-2-methyl-1,3-propanediyl ester; and octanal. Only four key odor-active compounds were identified from wood with waterborne coating: 2-methyl-propanoic acid, 1-(1,1-dimethylethyl)-2-methyl-1,3-propanediyl ester; 2-(2-hydroxypropoxy)-1-propanol; ethyl acetate; and copaene.

It was found that the intensities of 15 odor-active compounds decreased after lacquer treatment, namely, o-xylene, ethylbenzene, 1,3-dimethyl-benzene, benzaldehyde, 1-methylene-1H-indene, butylated hydroxytoluene, dibenzofuran, 2,6,6-trimethyl-(ñ)-bicyclo[3.1.1]hept-2-ene, limonene, hexanal, octanal, acetophenone, nonanal, decanal, and acetic acid. In contrast, the intensities of 3 odor-active compounds increased: acetophenone; 2-methyl-propanoic acid, 1-(1,1-dimethylethyl)-2-methyl-1,3-propanediyl ester; and ethanol. Another 15 odorants—namely, p-xylene; 1,2,4-trimethyl-benzene; 1,2,3-trimethyl-benzene; copaene; ethyl acetate; acetic acid, 2-methylpropyl ester; acetic acid, butyl ester; 2-pentanol, acetate; 1-methoxy-2-propyl acetate; pentanedioic acid, dimethyl ester; 2-butanol; 1-butanol; 1-methoxy-2-propanol; 2-pentanol; and 2-(2-hydroxypropoxy)-1-propanol—which were not detected in the solid wood, appeared after lacquer treatment.

Key odorous compound characteristics were identified as follows. Ethylbenzene was reported to be aromatic, as we reported by Larranaga et al*.*^31^ It has also been reported to have a sweet, gasoline-like odor in the CAMEO Chemicals hazardous material database^32^ and to have pungent character^33^. The present testing found 2,6,6-trimethyl-(ñ)-bicyclo[3.1.1]hept-2-ene had a pine-like and pungent odor, similar to the pine description reported in *Fenaroli’s Handbook of Flavor Ingredients*^34^, and a turpentine odor^35^. The limonene detected in this experiment had a fresh, lemon peel-like, pleasant characteristic, similar to the pleasant, lemon-like odor reported by O’Neil^36^; its odor has also been described as penetrating^37^. The 1,2,3-trimethyl-benzene in this experiment was reported to present an orchid candy-like character, whereas the U.S. National Institute for Occupational Safety and Health (NIOSH) reported a distinctive, aromatic odor^38^. The acetic acid, 2-methylpropyl ester showed fruit-like, flowery, and fresh characteristics, which is similar to the fruity and floral odor reported by NIOSH^39^. It was also reported to have a solvent, nail polish-like odor in research by Furia^40^. The acetic acid, butyl ester presented as fruity in this study; it has also been reported to have sweety odor characteristic^41^. 2-Pentanol, acetate left a fragrant and pleasant impression in this study and has also been found as fruity^42^. Octanal was reported as fruity and sour in this study, similar to the citrus-like odor reported by Panten et al*.*^43^; they found it also had a pungent character. The decanal detected in this experiment had a fresh mint-like characteristic, whereas its odor was described as citrus-like by Ashford^44^, orange peel-like by Kohlpaintner et al*.*^45^ and floral-fatty by Lewis^46^. The pentanedioic acid, dimethyl ester had a pleasant smell, similar to the faint agreeable odor reported in the Merck Index^47^.

Two odor-active compounds presented individual characteristics under different concentrations. Ethyl acetate was reported as fruity with the concentrations 248 µg/m^3^ of PU coating and 182 µg/m^3^ of waterborne coating, in accordance with the fruity odor reported by NIOSH^39^ and fruity with a brandy note by Fahlbusch et al*.*^48^ However, it was detected with an irritating, glue-like, pineapple-like, fragrant scent with a concentration of 6,007 µg/m^3^, similar to the fragrant characteristic provided by Sax^49^ and characteristic ether-like odor reminiscent of pineapple recorded in *Fenaroli’s Handbook of Flavor Ingredients*^50^. This phenomenon showed that the odor characteristic was related to its concentration; for the same substance, it may present different odor characteristics under different concentrations. Ethanol also showed individual characteristics with different concentrations. It presented alcohol-like with concentrations of 200 µg/m^3^ in PU coating, 649 µg/m^3^ in UV coating, and 109 µg/m^3^ in waterborne coating, whereas fragrant was showed with a concentration of 49 µg/m^3^ in solid wood. Five odorous compounds were detected by only a few researchers: 2-methyl-propanoic acid, 1-(1,1-dimethylethyl)-2-methyl-1,3-propanediyl ester (antiseptic solution); 2-(2-hydroxypropoxy)-1-propanol (cigarette-like); 1,3-dimethyl-benzene (metal-like); copaene (nut-like); and 1-methylene-1H-indene (wood-like).

### Comparison of odor characteristics from *Choerospondias axillaris* with different lacquer treatments

**Figure 3:**
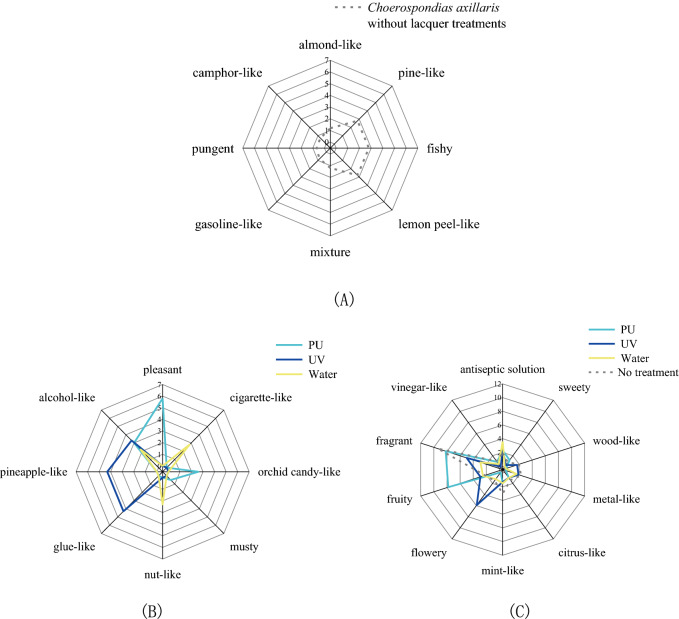
Changes of odor characteristics after lacquer treatment. (**A**) Odor characteristics disappeared after lacquer treatment. (**B**) Odor characteristics appeared after lacquer treatment. (**C**) Other changes to odor characteristics.

Figure 3 shows the changes in odor characteristics after lacquer treatment. It was found that PU, UV, and waterborne coatings had a good sealing effect on eight odor characteristics: almond-like, pine-like, fishy, lemon peel-like, mixture, gasoline-like, pungent, and camphor-like. These eight odor characteristics disappeared after lacquer treatment. At the same time, other characteristics, such as pleasant, cigarette-like, orchid candy-like, musty, nut-like, glue-like, pineapple-like, and alcohol-like, arose with the lacquer treatments. Among the newly added odor characteristics, PU coating had greater odor profiles of pleasant, orchid candy-like, and alcohol-like; the intensities were 5.8, 2.6, and 3.0, respectively. UV coating had greater odor profiles of glue-like, pineapple-like, and alcohol-like; the intensities of these three characteristics were 4.3, 4.3, and 3.3, respectively. The main characteristics of the newly added odors of the board with waterborne coating were cigarette-like (2.8), nut-like (2.3), and alcohol-like (2.2). Results showed the characteristic of alcohol-like increased in varying degrees after treatment by these three lacquers. The intensities of more odor characteristics changed in different degrees after treatment. After lacquer treatment, the total intensity of most odor characteristics, such as fragrant, vinegar-like, wood-like, metal-like, citrus-like, and mint-like, was reduced to a certain degree. It was found that the odor characteristic of fragrant was controlled well by UV and waterborne coatings; the odor intensities decreased 4.4 and 6.5, respectively. However, the UV coating increased the characteristic of flowery at the same time. The odor intensity of flowery rose by 4.4 after UV treatment. The characteristic of flowery could be inhibited by PU and waterborne coatings; the odor intensities decreased 1.5 and 0.3, respectively. The odor intensities of the fruity and antiseptic solution increased after coating with three lacquers. Among these, PU coating had a great influence on the characteristic of fruity. After finishing, its characteristic increased from 2.2 to 7.8, and the intensity increased by 5.6. The water-based coating has a great influence on the characteristics of disinfectant. After finishing, its waterborne coating increased from 1.9 to 3.6, and the intensity increased by 1.7.

For the wood with no treatment, the major impression was fragrant (9.4). The intensity of mint-like was 3.0. Lemon peel-like, pine-like, and fishy all had a rating of 2.8, followed by metal-like (2.3), fruity (2.2), and wood-like (2.0). The attributes antiseptic solution (1.9), flowery (1.5), citrus-like (1.4), almond-like (1.2), mixture (1.2), vinegar-like (1.0), gasoline-like (0.8), sweety (0.8), camphor-like (0.7) had lower intensities. Fragrant, fruity, and pleasant were the dominant odor characteristics of PU coating, with ratings of 8.2, 7.8, and 5.8, respectively, followed by alcohol-like (3.0), orchid candy-like (2.6), and antiseptic solution (2.3). The attributes mint-like (1.5), sweety (1.2), citrus-like (1.0), vinegar-like (0.9), and musty (0.5) were rated with low intensities. For UV coating, the dominant odor characteristics were flowery, fragrant, glue-like, and pineapple-like, with intensities of 5.9, 5.0, 4.3, and 4.3, respectively, followed by alcohol-like (3.3), fruity (2.9), and antiseptic solution (2.3). The attributes metal-like (1.9), wood-like (1.7), mint-like (1.3), citrus-like (1.0), and sweety (0.3) were rated with low intensities. Antiseptic solution (3.6), fragrant (2.9), cigarette-like (2.8), and fruity (2.5) were main profiles of the board with waterborne coating, followed by nut-like (2.3) and alcohol-like (2.2). The intensities of the characteristics of metal-like (1.6), mint-like (1.5), flowery (1.2), citrus-like (1.0), and vinegar-like (0.5) were relatively low.

### Multicomponent evaluation of *Choerospondias axillaris* with different lacquer treatments

**Figure 4:**
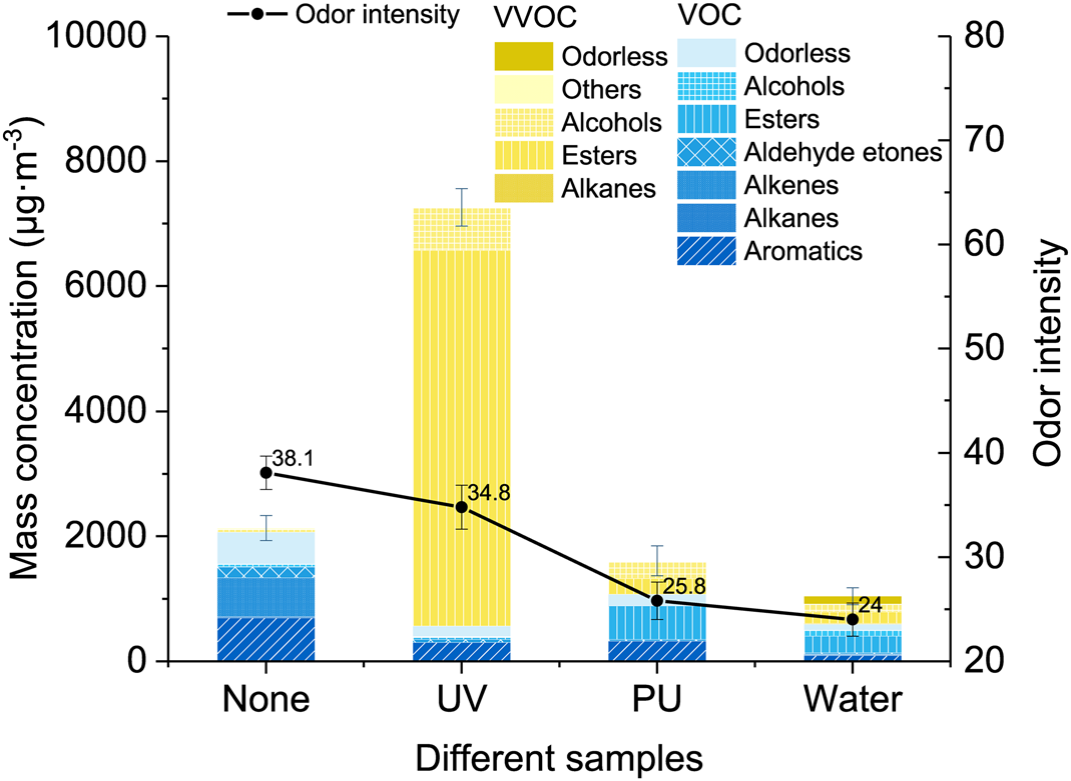
VOC and VVOC concentrations of different components and total odor intensity from *Choerospondias axillaris* with different lacquer treatments.

The odorous component concentrations of VOCs and VVOCs and OI rates from *Choerospondias axillaris* under different lacquer treatments are shown in Fig. 4. The sample with no treatment was used to compare the effect of lacquer treatments. The main odorous constituents from solid wood were aromatic, alkene, and aldehyde and ketone VOCs; only a few VVOCs that belong to alcohols and others were detected. After lacquer treatment, the aromatic VOC was still the important emission component, whereas the emission of alkene VOC and aldehyde and ketone VOC were inhibited and the release amount of VVOC (mainly esters and alcohols) increased. The total concentration (TC) of the UV coating was highest among these three lacquers, followed by PU and waterborne coatings. VOC was the main release of boards with PU, water, and no treatment; however, unlike other samples, the main odorous components of boards with UV coating were VVOCs, which account for 93.14% of TC. The main odorous constituents of UV were aromatic VOC, ester VVOC, and alcohol VVOC. The release of ester VVOC was mainly from ethyl acetate with a concentration of 6,007 µg/m^3^, which accounted for 83.59% of the TC. It may come from the solvent of UV coating and should be given close attention. For the board with PU and waterborne coatings, the main odorous constituents were aromatic VOC, ester VOC, alcohol VVOC, and ester VVOC.

After lacquer treatment, the OI of solid wood decreased. The OI of the board with no treatment presented the highest value (38.1), followed by PU (34.8), UV (25.80), and waterborne (24.00) coatings, which showed the odor from *Choerospondias axillaris* was inhibited in different degrees after lacquer treatment. Four samples were evaluated based on TVOC values on day 28 (TVOC_28_), TVVOC values on day 28 (TVVOC_28_), OI, *R*, and concentration of nonassessable compounds. The information was shown in Table 2. For TVOC_28_, it was found that the value of untreated solid wood was the highest among these four boards, with a mass concentration of 2066.19 µg/m^3^, followed by the wood with PU coating (1,072.27 µg/m^3^). The wood with UV coating (488.89 µg/m^3^) and waterborne coating (593.17 µg/m^3^) received a good evaluation, and the concentration was less than 1,000 µg/m^3^. However, TVVOC of UV coating (6,696.97 µg/m^3^) was significantly higher than that of PU coating (534.48 µg/m^3^), waterborne coating (451.71 µg/m^3^), and wood with no treatment (110.54 µg/m^3^). For the value of *R*, results showed that the value of UV coating was the highest (2.4246), which may cause by the high concentration of TVVOC. The *R* of solid wood was 1.6027, followed by the PU coating with 1.5218. The wood with waterborne coating had the lowest *R* of 0.3338.

**Table 2:** Evaluation index of *Choerospondias axillaris* with different lacquer treatments.

Index	UV	PU	Water	None
TVOC_28_ (µg/m^3^)	489	1,072	593	2066
TVVOC_28_ (µg/m^3^)	6,697	534	452	111
OI	25.80	34.80	24.00	38.10
R (assessable compound: all VVOC and VOC with LCI)	2.4246	1.5218	0.3338	1.6027
Concentration of non-assessable compounds (µg/m3) (sum of VOC and VVOC with unknown LCI)	89	28	449	608

The study showed that to assess emission from materials more comprehensively, it is not enough evaluate the concentration of VOCs. For some boards with UV treatment, the evaluation was good when referring to the value of TVOC_28_ and OI. However, when the *R* value and TVVOC were considered, the evaluation results changed. Based on the index of different boards, the wood with waterborne coating was the best choice among these three boards, with the lowest index of TVOC_28_, TVVOC_28_, OI, and *R*. However, the concentration of nonassessable compounds was higher than that of the wood with PU and UV, which should be given attention.

## Conclusion

In the present study, odor-active compounds and odor characteristics from *Choerospondias axillaris* with different moisture content percentages and lacquer treatments were investigated. A multicomponent evaluation method was used to evaluate the health risks of the boards. The results of this study can be used to evaluate the wooden materials. Meanwhile, this study is helpful in establishing an odor database of solid wood and wood with lacquer treatments.

Alcohols and alkenes were the main release components of *Choerospondias axillaris*, followed by aromatics, aldehydes and ketones, and esters. In total, 11 key odor-active compounds were identified as moisture content decreased gradually, concentrating between 15 and 33 min in GC-O. The odor intensity of benzaldehyde, dibenzofuran, octanal, ethanol, and 2-ethyl-1-hexanol, 2,6,6-trimethyl-(ñ)-bicyclo[3.1.1]hept-2-ene, limonene, and decanal presented a downward trend as moisture content decreased. Moisture content had a significant effect on emissions from *Choerospondias axillaris*. TVOC, TVVOC, and OI rates reached their maximum values when the moisture content was 60% and then decreased as the moisture content decreased.

In total, 35 odor-active compounds were identified in the odor control list of *Choerospondias axillaris* with different lacquer treatments. It was shown that PU, UV, and waterborne coatings had a good inhibitory effect on the characteristics of almond-like, pine-like, fishy, lemon peel-like, mixture, gasoline-like, pungent, and camphor-like, whereas scents of cigarette-like, orchid candy-like, musty, nut-like, glue-like, pineapple-like, and alcohol-like arose after lacquer treatment. The TC of UV coating was highest among the three lacquers, followed by PU and waterborne coatings. VOC was the main release of boards with PU, water, and no treatment, whereas the main odorous components of boards with UV treatment were VVOC, which were mainly from the ethyl acetate and should be given close attention. Based on the multicomponent evaluation method, which considers TVOC_28_, TVVOC_28_, OI, *R*, and concentration of nonassessable compounds, the wood with waterborne coating proved to be the best choice among these three boards. However, the concentration of nonassessable compounds within the board with waterborne coating was higher than those with UV and PU coatings.

## Methods and materials

### Materials

The wood of *Choerospondias axillaris* (Roxb.) Burtt et Hill produced in GuangYun Forest Farm (Guilin City, Guangxi, China) was used in this experiment. The samples were cut into round pieces (60 mm diameter and 16 mm thickness), with an exposed area of 5.65 × 10^−3^ m^2^. The sample was gradually dried to moisture content percentages of 60% ± 2%, 45% ± 2%, 30% ± 2%, 10% ± 2% (equilibrium moisture content), and 5% ± 2% using a bake-out furnace with a temperature of 45 °C ± 1 °C. The same sample was used in each group of measurements. The wood (with a moisture content of 10% ± 2%) was lacquered using PU, waterborne, and UV-curable coatings. The parameters were as follows. PU coating: Huarun, transparent primer/matte finish (main and curing agent); diluent = 2:1:1; painted two primers (10 m^2^/kg/session) and painted two finish layers (10 m^2^/kg/session), with 12 h between painting sessions. Waterborne coating: Sankeshu 360 waterborne wood paint, transparent primer/varnish finish, and distilled water (main agent); distilled water = 10:1; painted two primers (10 m^2^/kg/session) and two finish layers (10 m^2^/kg/session), with 12 h between painting sessions. UV-curable coating: Sujinghuaxue; painted twice (10 m^2^/kg/session) and sprayed after cleaning the spray gun and product surface, with 3–10 min of UV curing (at 55 °C). The painting environment conditions were as follows: indoor temperature, 23 °C ± 2 °C; relative humidity, 40% ± 10%. The room was in a continuous ventilation state. The surface of the solid wood was polished with 150-mesh sandpaper, and 180-mesh sandpaper was used between painting sessions. After lacquer treatment, the edges of the specimens were wrapped with aluminum foil to prevent the release of compounds, the samples were vacuum stored in polytetrafluoroethylene (PTFE) bags and refrigerated until needed.

### Sampling

VOCs and VVOCs (2 L) from the sample was adsorbed using a microchamber/thermal extractor, which could be adjusted from 0 to 250 °C. The cell volume was 1.35 × 10^−4^ m^3^, and the loading rate (the ratio of the panel area to the microchamber volume) was 41.85 m^2^/m^3^. Purified humidified air was supplied throughout the experiment. The environment conditions were as follows: temperature, 23 °C ± 2 °C; relative humidity, 40% ± 10%; ratio of air exchange rate to loading factor, 0.5 m^3^ m^−2^ h^−1^. Two types of tubes were used in this experiment: a Tenax-TA tube and tubes with multisorbents of carbopack C, carbopack B, and carboxen 1000 (Markes International, South Wales, UK).

The emissions from *Choerospondias axillaris* samples with different percentages of moisture content were collected as soon as the wood was dried to the specified moisture content, whereas *Choerospondias axillaris* samples under different lacquer treatments were assessed after 28 days for long-term behavior of VOC and VVOC emissions. After sampling, the tubes were wrapped in PTFE bags until needed.

### VOC and VVOC analytical methodology

VOC and VVOC release from samples was analyzed by TD–GC–MS/O. The emissions were identified compared with the MS spectra from the National Institute of Standards and Technology (NIST) and Wiley MS libraries, which matched degrees up to 800 or more. The compounds were quantified according to the Chinese national standard GB/T 29899-2013^51^.

The DSQ II series quadrupole gas chromatography–mass spectroscopy (GC–MS) unit came from Thermo Fisher Scientific (Schwerte, Germany). Chromatography was performed with a DB-5 quartz capillary column (30,000 m long × 0.26 mm inner diameter × 0.25 µm particle size; Agilent Technologies, Santa Clara, CA). The parameters were as follows: cold-trap adsorption temperature, − 15 °C; thermal desorption temperature, 280 °C; thermal analysis time, 10 min; injection time, 1 min. Helium was used as the carrier gas. The chromatographic column was initially kept at 40 °C for 2 min, and then the temperature was increased to 50 °C (in 2 °C min^−1^ increments) and held at that temperature for 4 min. Finally, the temperature was increased to 250 °C in 10 °C min^−1^ increments and held for 8 min, with the injection port temperature also at 250 °C.

The parameters of GC–MS conditions were as follows: ionization mode, electron ionization; ion energy, 70 eV; ion source temperature, 230 °C; transmission line temperature, 270 °C; mass scan range 50–650 atomic mass units.

### Analytical odor technology: GC–O

GC-O technology was used in this experiment combined with GC–MS. The Sniffer 9,100 Olfactory Detector came from Brechbühler (Echallens, Switzerland). The transmission line temperature was 150 °C, and nitrogen was used as the carrier gas through a purge valve. Moist air was added to prevent dehydration of the nasal mucosa of the odor assessors. Direct intensity methods were chosen for the analysis of the compounds.

The test procedure was set according to Wang et al*.*^52^ Based on specific screening and training recommendations in ISO 12219-7, four assessors (between 20 and 30 years old, with no history of smoking and no olfactory organ disease) were chosen to form an odor-analysis evaluation group. The experimental environment was set to National Standards Authority of Ireland reference standard EN 13725-2003. The room was well ventilated, and there were no peculiar smells within the room. The temperature was kept at 23 °C ± 2 °C throughout the experiment. Activities such as eating, which might affect indoor odors, were forbidden for 5 h before the experiment. During a GC run (described earlier), the human sensory-evaluation assessors recorded the odorants by characteristic and intensity value, as well as the retention time. The detection time for each sample lasted about 50 min. A six-point scale ranging from 0 to 5 was used for intensity judgment according to Japanese Ministry of the Environment standards: 0 = none, 1 = very weak, 2 = weak, 3 = moderate, 4 = strong, and 5 = very strong. The fingerprint span method was used simultaneously to verify the results. Experimental results were recorded when the same odor characteristics were described by at least two assessors. Through a Microsoft Excel data processing system, the relative percentage content of each chemical component in wood odorous substances was obtained by the area normalization method. The compounds were identified by aroma recognition and odor description. The intensity value was based on the average values from the different assessors. A compound’s refractive index value was calculated by the retention time of n-alkane (C_6_–C_30_) under the same conditions. The identification of odorous compounds was based on GC-O and compared with the literature.

### Risk assessment method

Based on Report 19 of the European Collaborative Action on Indoor Air Quality and its Impact on Man, TVOC can be used to define the concentration of VOCs if there is a VOC mixture in indoor air^53^. According to this definition, the total concentration of VVOCs was expressed as TVVOC, the total concentration of VOCs and VVOCs was expressed as TC, and the overall odor intensity was expressed as the total odor intensity (OI).

The lowest concentration of interest (LCI) is an evaluation level above which, according to the best professional judgment, the pollutant may have some effect on people in the indoor environment^54^. Substances whose concentrations in the test chamber air exceed 5 µg/m^3^ are evaluated based on LCI. They can be quantified using their individual calibration factors. For the evaluation of each compound *i*, the ratio *R*_*i*_ is established as defined in Eq. (2)^55^:

2 $$R_{i} = C_{i} /{\text{LCI}}_{i}$$

where *C*_*i*_ is the chamber concentration of compound *i*. For *R*_*i*_ < 1, it is assumed that there will be no effects. If several compounds with a concentration > 5 µg/m^3^ are detected, additive effects are assumed, and then *R*, the sum of all *R*_*i*_, will not exceed the value 1.

Because of a lack of adequate studies and experimental data, the European Union LCI value of some chemicals cannot be derived directly. In this case, a hazard assessment may rely upon predictive approaches, such as read across and grouping of substances^56^. If test data are available for a range of chemicals with a closely related structure, it is possible to extrapolate, with confidence, from data-rich compounds to data-poor compounds. Because subtle changes in chemical structure can have a significant effect on biological activity, especially if toxicity is mediated by binding to a receptor, certain minimum criteria need to be considered when undertaking a hazard assessment using predictive approaches. However, for some compounds, the LCI cannot be determined even using the method of read across. In this case, the components were calculated for those VOCs and VVOCs with a nonassessable LCI value to avoid the risk of a positive evaluation of a product that emits larger quantities of nonassessable substances.

**Figure 5:**
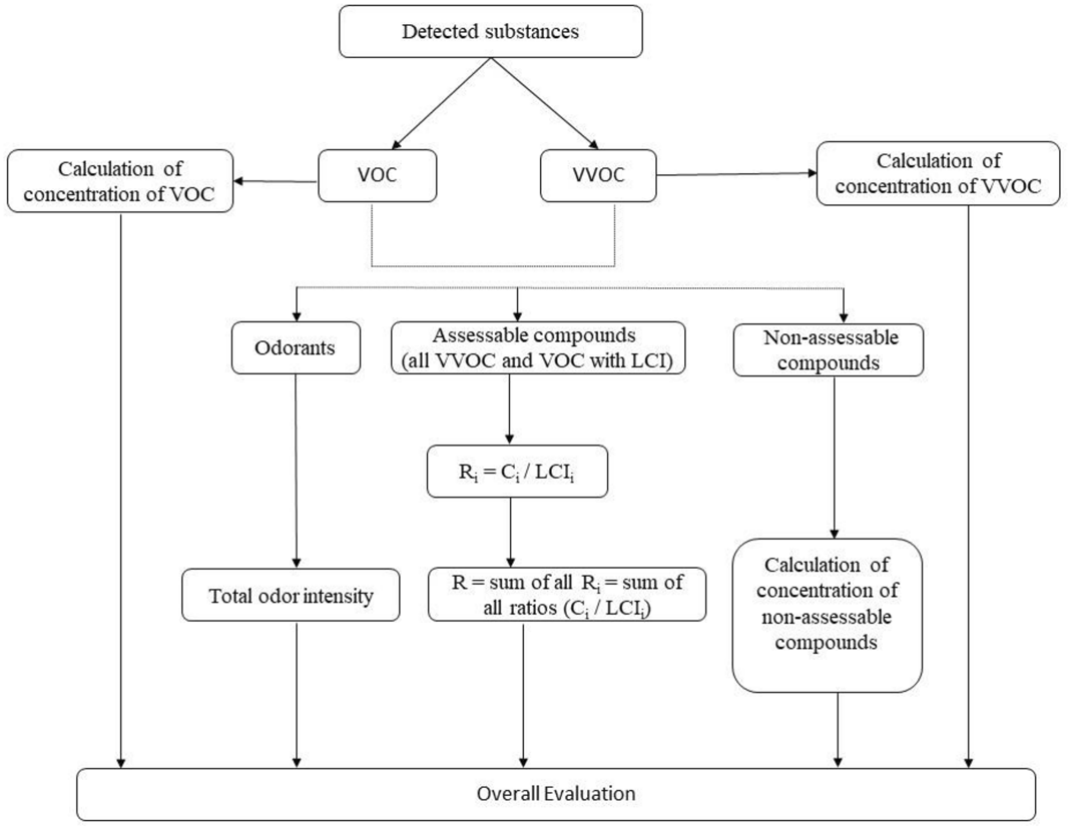
Principles for evaluation of emission.

Referring to the Health-Related Evaluation Procedure for VOC Emissions from Building Products standard from AgBB, the evaluation in this experiment was based on the TVOC and TVVOC values on day 28, total odor intensity, *R*, and concentration of nonassessable compounds. The process for evaluation of emission is shown in Fig. 5.
